# Older Adults’ Preference for Landscape Features Along Urban Park Walkways in Nanjing, China

**DOI:** 10.3390/ijerph16203808

**Published:** 2019-10-10

**Authors:** Xinxin Wang, Susan Rodiek

**Affiliations:** 1 Department of Landscape Architecture, College of Horticulture, Post-doctoral Research Station in Public Administration, Nanjing Agricultural University, Nanjing 210095, China; 2 Department of Architecture, Center for Health Systems & Design, Texas A&M University, College Station, TX 77843-3137, USA; rodiek@tamu.edu

**Keywords:** outdoor usage, elderly, seniors, aging, photographic comparison, physical activity, health and well-being

## Abstract

Evidence shows that walking in urban parks has multiple health benefits for older adults, but little research is available on their preference for specific walkway features. This study explored a range of common landscape and hardscape features to learn which were preferred by park users over age 60. This photo comparison study hypothesized that older adults would prefer certain features of urban park walkways, with each feature represented by four different paired images (28 pairs in all). Within each pair of photos, both were identical except for the specific feature being tested in that comparison, where the image was digitally modified to depict the hypothesized feature. A total of 283 older adults (mean age 71 years) completed the survey by selecting the images they preferred. In this Chinese sample, older park users significantly favored all seven hypothesized walkway features, providing empirical support for the existing research and design-based literature on green space for older adults. This study found minor gender differences in visual preferences for walkway features and increasing preference for access to seating with advancing age. By helping to confirm which walkway features are preferred by older adults, these findings can be used to improve the future design and management of urban parks in China, which are an important source of exercise and recreation for nearby elderly residents.

## 1. Introduction

### 1.1. Urban Parks as Places for Walking

Urban parks provide ideal places for older city-dwellers to rest and relax, and more importantly to engage in outdoor activities and social contact [1]. Due to declining physical strength and sensory acuity, older people typically have reduced mobility, and often prefer green spaces that are easily accessible from their homes. Older adults in China, especially those living in high-density cities, have been found to spend substantial amounts of time in urban parks [2]. Studies conducted in Taipei and Nanchang found that *more than half (61% and 54%) of urban park users were older adults* [3,4]. In Shanghai, a behavior-mapping study in three small urban parks found that *over 80% of park users were retired older adults* during weekdays [5]. 

As a moderately intense exercise, walking is the most common behavior in urban parks, and can be easily integrated into other activities. Walking has been reported to have a greater appeal for older adults than high-intensity exercise [6]. For older adults, regular walking may strengthen muscles, reduce the risk of falling, alleviate depression, improve sleep patterns, and improve overall quality of life [7,8]. 

Although evidence shows that urban parks can contribute to physical activity, parks and walkways may be underutilized if they are poorly designed or maintained [9,10,11]. Since park visitors may perceive the landscape differently from park designers, it is important for designers, planners, and park managers to understand the landscape characteristics that influence the walking experience of older users [12]. By incorporating evidence based on research, specific landscape plans and designs can be created to accommodate older people’s behavioral needs.

### 1.2. Relevant Studies

Although many studies have found correlations between walkways and physical activity, very few have analyzed the specific design and landscape features associated with walkways. Using a visual discrete choice experiment, Arnberger et al. analyzed the heat-adjusted designs in green spaces for older adults [13]. Alves et al. examined the environmental attributes relevant to preference for a neighborhood park [14]. Although these studies suggest that older adults’ walking behaviors were influenced by the overall urban park landscape characteristics, little was learned about older adults’ preference for specific landscape features along the walkways, particularly in terms of preferred plantings and views.

In China, only a limited number of studies have examined the landscape features of urban park walkways from the perspective of older adults. Using space syntax analysis, Zhai and Baran analyzed walkway configurations and older persons’ walking behaviors in urban parks [15]. The same authors also related design characteristics of walkways to the numbers of older persons observed on-site [16]. Duan et al. investigated older people’s park use in Hong Kong, China and Leipzig, Germany [17]. This study found that urban park walkways were the most heavily-used physical activity areas by older adults in both cities; however, the study focused more on the use of recreational facilities, rather than preferences in landscape perception. Due to intense urbanization and a rapidly-aging population in China, it is becoming increasingly important to plan and design urban parks based on the landscape preferences of older adults in Chinese cities.

### 1.3. Environmental Characteristics and Features

Studies with older adults have found that specific landscape characteristics can enhance walking activities in residential neighborhoods [18], local street environments [19], and urban parks [20]. Certain landscape attributes of urban parks have been analyzed using choice-based conjoint analysis, indicating that older adults prefer amenities such as toilets, shade structures, well-paved surfaces, trees and plants, minimal traffic, and seating along walkways [14,21]. Although studies have gleaned information about which landscape features are important to older users, they did not examine specifically how these features should be designed and arranged [22]. For example, a study found that generally “trees/plants” were highly preferred by older adults but did not address the types and preferred arrangements of these plants, when viewed from walkways [23]. Similarly, although park seating was found to be strongly preferred by older adults, few studies have differentiated among different types of seating, such as emphasizing benches with backrests and/or armrests to support frail older users [24]. 

This study fills an existing gap in the literature by empirically investigating several common landscape features of urban park walkways in China, to better understand older adults’ preferences that may potentially impact their walking and outdoor usage. Although large- and small-scale features such as nighttime lighting, walkway layout, and paving conditions have been found to influence park usage, the present study was focused primarily on meso-scale features such as landscape plantings and hardscape elements such as benches, which were considered to be more feasible to test with photographic comparison.

To be eligible for inclusion in this study, landscape features were required to meet the following criteria:

(a) To be visible from the park walkways;
(b) To be tangible, physical features that could feasibly be addressed in the design or renovation of urban parks in China;
(c) To have emerged in the existing literature as important to older adults;
(d) To include both *magnets* (attractive features expected to *encourage* usage) and *barriers* (elements expected to *discourage* usage).

Among the walkway features that met the above criteria, the following emerged as important, based on the literature and their suitability for photo comparison:

1. *Ground cover plants*: covered with grass or other plants, instead of bare land.
2. *Colorful flowers*: having decorative flowers or blooming trees along the walkway.
3. *Diverse mix of plants*: having different heights and types of plants to increase spatial hierarchy and visual/olfactory stimuli.
4. *Visual accessibility*: providing non-obstructed views along the walkway, without plants blocking the view.
5. *Canopy trees*: having taller trees that provide shade and a sense of enclosure for walkers.
6. *Available seating*: usable seating is located beside the walkway.
7. *Bench with backs and arms*: seating provides backrests and/or armrests for support.

These features are prominent in published design recommendations for older adults, and in empirical studies:

*Ground cover plants*, which are short enough to walk across, were the most common components of green spaces in many cities worldwide [25]. Ground cover plants have been linked to participation in sports and increased social interaction [26,27]; ground cover plants were preferred by older adults in a previous photo comparison study [28] and in semi-structured interviews [14]. 

*Colorful flowers* are widely reported as being very important to older adults in design-based literature and preference-based studies. They were strongly preferred by assisted living residents in the United States [29], elderly visitors of public parks in China [20], and residents of nursing homes in Sweden [30]. Colorful flowers were noted to increase older adults’ sensory enjoyment of nature [31,32].

A *diverse mix of plants* can provide abundant greenery, and plant materials at different heights can support horticultural therapy and allow older adults closer contact with nature [30,33,34,35]. Research has found that older adults preferred densely and diversely vegetated green space [36,37]. Studies have shown improved physiological and psychological measures associated with time spent in outdoor green spaces with a diverse mix of plants [28,38].

*Visual accessibility* can be psychologically satisfying by providing a distant vista [39]; studies in the general population found that views of natural settings had remarkable effects on reducing stress [40]. For older people, access to views has been associated with stronger preference and higher levels of outdoor usage [41,42]. 

*Canopy trees* are tall enough to see under, and typically provide overhead enclosure and shade. The ‘prospect-refuge theory’ and the ‘savanna hypothesis’ both suggest that humans are attracted to canopy trees because they are optimal for survival [39,43]; later studies have confirmed this preference [44]. For older adults, shade trees can improve outdoor comfort, reduce glare, and serve as landmarks in outdoor areas [45,46,47]. 

Having *available* *seating* can provide places for older adults to rest, watch people, and enjoy nature [34,46,48]. Design guidelines have noted that benches along the walkway can encourage exercise by residents in nursing homes [49], in low-cost housing for the elderly [42], and in the city streets [50]. Seating places were preferred by long-term care residents, especially those with physical limitations [36].

*Benches with backs and arms* have been identified as important amenities in an effective healing garden [51]; they provide support while people sit down and rise up, enhancing safety and accessibility for frail older park users [52]. Precautionary designs to help older people feel comfortable outdoors are emphasized by previous research [41,53,54]; replacing existing benches with those with backs and arms is a relatively easy and inexpensive way to improve ease of usage for older adults.

## 2. Methods

### 2.1. Overview

This study employed photographic comparison methods with older park users in Nanjing, China, using printed booklets. The following sections describe the site location and selection, production of photographic images, and the data collection process.

### 2.2. Site Selection

*City and districts.* Nanjing City is the capital of Jiangsu Province in the Yangtze River Delta region of eastern China, with a humid subtropical climate and four distinct seasons [55]. In 2017, the population was 8.34 million, with 16.5% aged 60 or over. The high-density urban area of Nanjing contains four administrative districts: Xuanwu, Qinhuai, Gulou, and Jianye. 

The four districts are roughly similar in terms of cultural and socio-economic backgrounds; over 98% of the population in each district are Han Chinese, with similar average disposable incomes, according to the Bureau of Statistics of Nanjing [56]. Xuanwu District, used in this study, had the second-largest geographical area (75.46 km^2^), and the third-largest resident population (634,300), with a population density of over 8000 people per square kilometer in 2016 [57]. 

**Figure 1:**
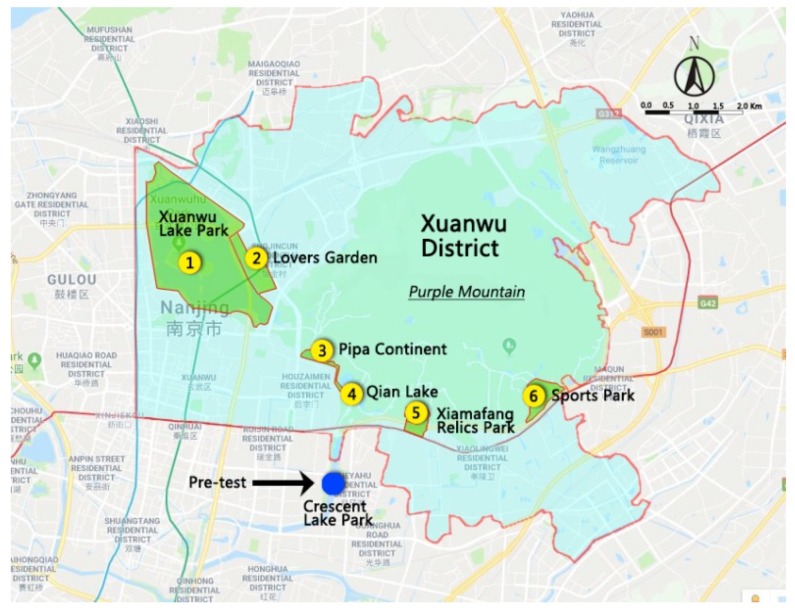
Urban parks in the Xuanwu District, Nanjing City (Source: Google Maps).

*Xuanwu District parks*. Within the Xuanwu District, all six urban parks with free admission and good transport accessibility (shown in Figure 1) were included in the data collection phase of the study: 1) Xuanwu Lake Park, 2) Lovers Garden, 3) Pipa Continent, 4) Qian Lake, 5) Xiamafang Relics Park, and 6) Sports Park. A few other parks exist on Purple Mountain, but some are reserved for resource conservation, while the rest either charge an admission fee or are located far from residential areas. 

**Figure 2:**
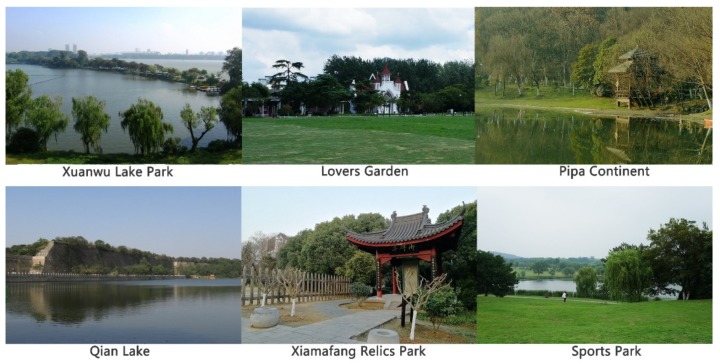
Photos of the six urban parks included in the study.

*Parks used in this study.* Collectively, the six parks used in this study *(*Figure 2) had substantial diversity and attracted users from a wide range of residential areas. “Xuanwu Lake Park” is the largest park in the district and is located in the highest density area. It was once an imperial lake garden and is now an urban park with five islands in the lake. “Lovers Garden” has European-style flowerbeds along the walkways and is a popular spot for wedding photographs. The other four parks in the Xuanwu District are located at the south foot of Purple Mountain. “Pipa Continent” has waterfront landings around Pipa Lake, and a view of the city walls from the Ming Dynasty. “Qian Lake” is a linear park along the Ming city walls, where people can enjoy the lake scenery along the walkways. “Xiamafang Relics Park” was built near the Ming Tomb; visitors can see outdoor exhibits of ancient remains. “Sports Park” is a newly built urban park with gently rolling topography, large lawns, and a distant view of Purple Mountain. In addition to the included parks, another nearby park, “Crescent Lake Park,” just outside the Xuanwu District, was used to pre-test the instrument and study protocol.

### 2.3. Photographic Comparison Method Used

This study used printed color booklets of photographic images to obtain preference feedback from park users; matched pairs of photos used digital manipulation to isolate the features of interest. This method has proved to be a helpful tool to communicate with stakeholders and assess visual preferences for alternative designs [28,58,59,60], and was reported to be especially effective with older adults [61,62]. 

### 2.4. Producing Photographic Images and Booklet

All 31 urban parks included in the high-density areas of Nanjing were visited, and more than 600 photographs were taken between August and October 2017. The type and location of photos depended on scenic qualities and the landscape features to be tested. Most photographs were taken when standing in the middle of the walkway—the pavement was placed in the center and with the green space along the walkway on both sides, providing a balanced view and an immersive experience of taking a walk. In order to capture more detail and minimize strong shadows, photos were typically taken in overcast weather with soft, low-light conditions. In addition to photos of walkways, other landscape photos were also taken in the urban parks, as materials and textures for photo editing purposes.

The primary researcher first reviewed the photographs and proposed a selection that: (a) represented typical urban park walkways in this region, with common width and curves; (b) clearly displayed (or lacked) the hypothesized landscape features, and (c) could feasibly be edited by adding or removing the features of interest. These photos were then reviewed by the secondary researcher, and a reiterative process followed, with both researchers analyzing the merits of individual photos, until a consensus was reached on which ones to include in the study. The selected photos were then digitally edited using Adobe Photoshop CS6 (Adobe, San Jose, CA, USA).

**Figure 3:**
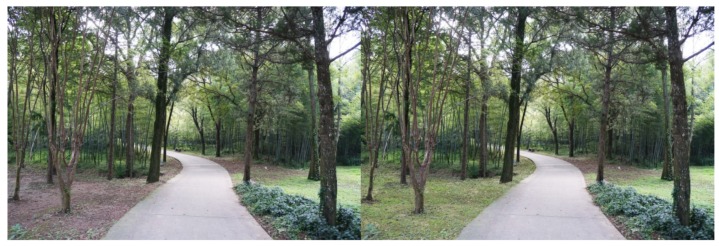
Example: Ground cover plants (grass of similar texture was added to the side with bare ground).

**Figure 4:**
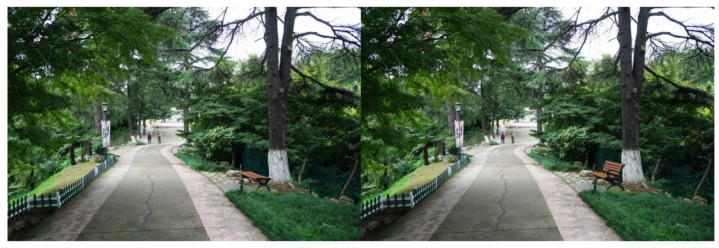
Example: Bench with backs and arms (a bench without backs or arms was replaced by a similar bench having both).

**Figure 5:**
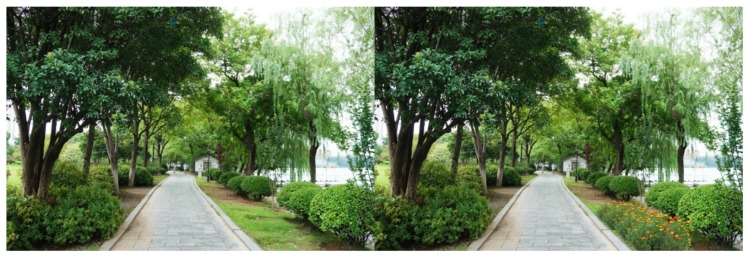
Example: Colorful flowers (herbaceous flowers were added).

**Figure 6:**
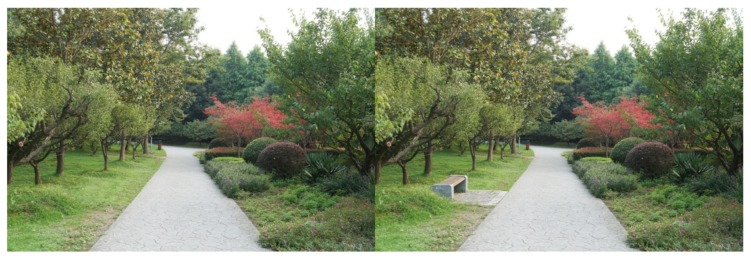
Example: Available seating (a bench and paved access pad were removed).

**Figure 7:**
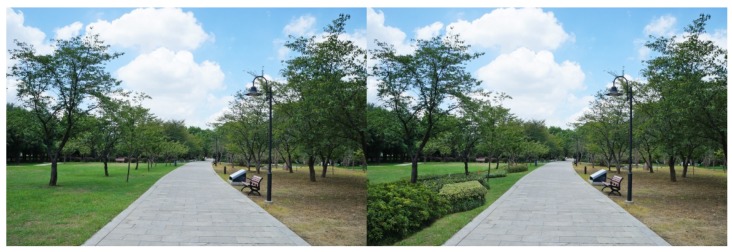
Example: Diverse mix of plants (“barrier” height shrubs were added).

**Figure 8:**
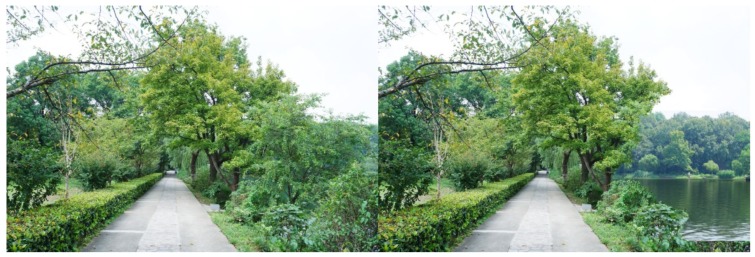
Example: Visual accessibility (“screening” height shrubs in front were partly removed, to reveal water view beyond).

**Figure 9:**
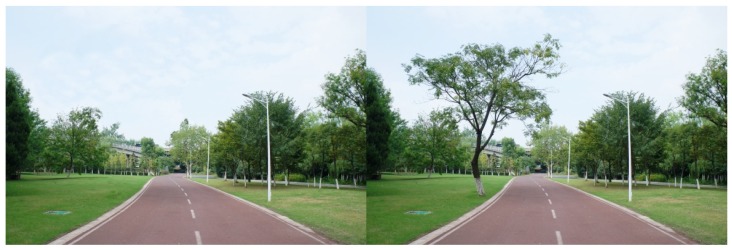
Example: Canopy trees (the overhead canopy tree was removed).

For each tested feature, four pairs of photos were produced to make comparisons. Half of the original photos were modified by *adding* the landscape features, and the other half by removing the features. As there were seven features tested in this study, a total of 28 pairs of images were produced. Both researchers carefully examined the final photos, to make sure the differences of the paired photos could be easily recognized, but the digital revision artifacts would not be obvious to viewers. The photo booklets used by all study participants included the same set of 28 paired images, shown in Appendix A, Table A1. Figure 3, Figure 4, Figure 5, Figure 6, Figure 7, Figure 8 and Figure 9 show seven pairs of images, each presenting a single example of each of the seven features tested. To make it easier for readers, all figures in this paper are arranged to show the hypothetically-preferred image at the right side.

To increase participants’ visual focus on the images, a 2 cm black border was shown around each photo, as done by Rodiek and Fried [28]. Before printing, we rearranged the paired photos in random order. Half of the pairs were also randomly selected to have the hypothesized preferred images placed on the left side, and the other half placed on the right side. In addition, the overall order of the photos was reversed in half of the booklets. Photos were color printed in landscape mode on A4 heavyweight matte papers and spiral bound by a local printing service.

### 2.5. Pre-Test Instrument 

**Figure 10:**
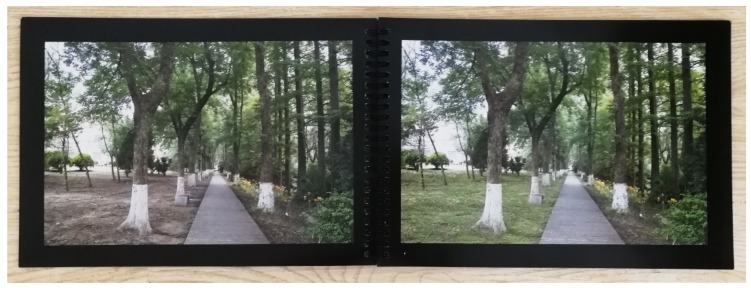
Photo of the booklet in side-by-side format.

Before the data collection phase began, the preliminary instrument (printed color booklet) was pre-tested at a nearby park not included in the study (see Figure 1), in order to verify the preferred booklet format, test the field protocol, and ascertain whether any images needed adjustment. The older adults participating in the pre-test seemed to easily be able to select the photos they preferred. Based on participants’ comments, a few improvements were made in the prepared booklet. Most participants preferred the booklets in *side-by-side format* (Figure 10) rather than *one above another*, saying they were more convenient and easier to use. Some said the plastic covers of the booklets were slippery to hold or put on their lap, so we added rough paperboards over the covers. In addition to overall quality control, a few revisions were made to individual images. In one original photo, a large blue tree label was hanging in the tree, which distracted participants’ attention from other landscape features. The blue label was removed from the photographs, as it was not a common or necessary element in urban park walkways. In another photo, the added shrubs were slightly dark and difficult to see, so the plants were brightened. Data from the pre-test was not included in the main study.

### 2.6. Data Collection Phase

**Figure 11:**
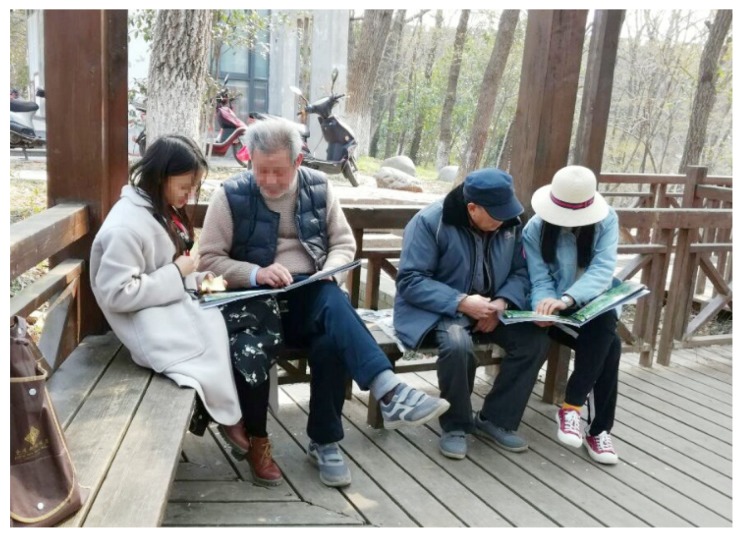
Participants did surveys in a pavilion along the walkway, together with the research assistants (faces were modified with a mosaic to protect privacy).

Recruiting participants. This study targeted adults aged 60 or older, who were local residents using the urban park in their daily lives; tourists coming from other areas were not included in the survey. Conducting the survey in park settings made it possible to directly recruit older adults who were demonstrated users of urban parks. Several research assistants (university students) were trained in data collection procedures. This project was sponsored by the College of Horticulture, Nanjing Agricultural University, and informed consent was obtained from all participants. In each of the six parks used in the study, one to four survey points were chosen, depending on the size of the park. Every survey point was along the main walkway and had a rest facility such as a pergola or pavilion, where participants could sit to complete the survey (Figure 11). Investigators were standing at the designated survey point in the parks. They approached the first person coming by who looked old enough and explained the purpose of the survey to them. Those who agreed to participate and met the study inclusion criteria (local residents over 60 years old) were invited to sit in the nearby rest facility to take the survey. As soon as the participant had finished, the research assistant made any notes necessary, and then waited for five minutes before asking another person to participate. To avoid having the survey appear to be a commercial propaganda activity, participants were not offered any gifts or payments.

Data collection protocol. The survey was conducted on sunny days between March and April 2018, from 7–11 am and 1–5 pm. The research assistants handed out one of two forms of the booklet (with page order reversed) to participants. Both forms of the booklet contained the same 28 pairs of images. They mentioned that the two photos in each pair were identical except for one of the features, and that all the photos had been taken in Nanjing urban parks. Older adults were asked to imagine themselves walking in the park, and to *select the image of the walkway they “preferred to use.”* They were shown how to select their preferred image in each pair by applying sticky notes to the one they selected. When the older adults finished their selections, investigators asked their specific age, and checked the booklet, to make sure one image had been selected in each pair. After the participant left, the investigator wrote down the response data using code numbers, and then returned the booklet to its original condition. 

### 2.7. Data Analysis

The age and gender of the participants were manually entered in Excel worksheets, along with their survey responses. The measure for landscape features older adults preferred to use was calculated as the percentage of the example pairs where the hypothesized photo was selected, taking on possible values of 0%, 25%, 50%, 75%, or 100%. It is worth noting that unlike Likert-type measures of preference, these scales were of ratio/numeric status. A one-sample *t*-test was used to determine the preference of each landscape characteristic and the feature examples. Paired *t*-tests were conducted in analyzing the differences between environmental features. An independent samples test was used to analyze the differences between the genders. The relationship between features and age was calculated by Spearman correlation. The level of significance was set at *p* < 0.05. The statistical analysis was completed in IBM SPSS Statistics 19 (SPSS Inc., Chicago, IL, USA).

## 3. Results

### 3.1. Demographic Information

As shown in Table 1, data from a total of 283 older adults (mean 70.93 years, standard deviation (SD) 6.88) was analyzed in this survey; 176 were males (62.19%) and 107 were females (37.81%). More participants were surveyed in Xuanwu Lake Park and Lovers Garden, because the parks were larger and had more visitors. The mean ages of the participants were similar across the different urban parks. For every park, there were more male participants than females, with the highest percentage of males at Xuanwu Lake Park.

The mean rejection rate was 34.94% and was fairly similar across all the parks; the main reasons given for rejection were because they were busy, or just unwilling to participate in a survey. A few of the accompanied older adults did not hear or see well, so their younger partners rejected our invitations. Another 22 people did not fully complete the selection, and their data was not included in the final analyzed data. Since older adults can be skeptical about providing personal information to strangers in a public place, they were not asked about their educational or economic backgrounds.

### 3.2. Preference for the Hypothesized Features

#### 3.2.1. Preferred Landscape Features

**Figure 12:**
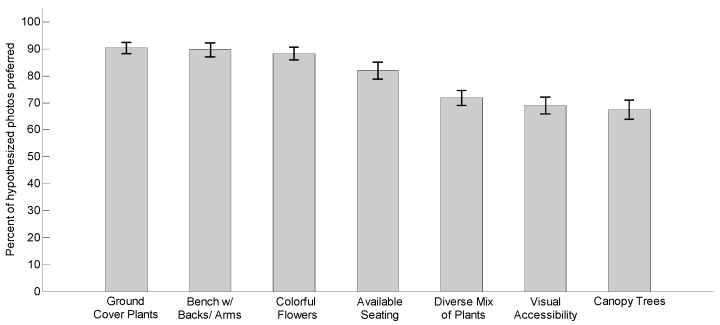
Mean for hypothetically preferred features with 95% confidence interval error bars.

This study investigated whether or not the subjects preferred the images depicting the hypothesized features. As each feature was represented by four different example photos, the percentage of the hypothesized photos selected by the subjects was calculated to measure preference intensity. A one sample *t*-test was used to compare the preference percentage to a 50% test value. As shown in Table 2 and Figure 12, the measures of preference ranged from an average of 67.40% to 90.37%. All seven features showed significant differences (*p* < 0.001), indicating that all the hypothesized features were preferred by participants.

#### 3.2.2. Differences Between the Features

Paired *t*-tests were used to determine whether subjects preferred to use certain walkway features more than others. Table 3 compares the differences between preference measures of different features. The subjects especially preferred walkway features such as “Ground cover plants” (90.37%), “Bench with backs and arms” (89.66%), and “Colorful flowers” (88.25%), more than the other four features (*p* ≤ 0.001). It is worth noting that “Bench with backs and arms” was more highly preferred than “Available seating” (*p* < 0.001) by older adults. The feature “Available seating” (81.89%) was more preferred than the features including “Diverse mix of plants” (71.82%), followed by “Visual accessibility” (68.99%), and “Canopy trees” (67.40%).

#### 3.2.3. Image Examples

**Figure 13:**
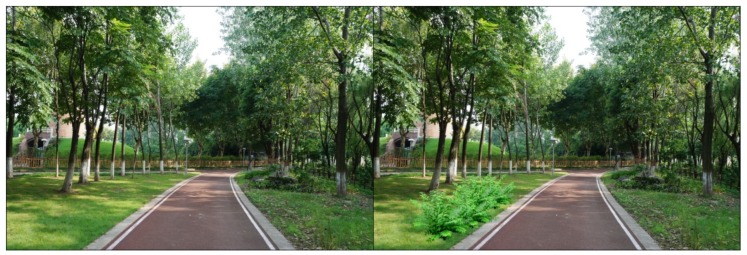
Diverse mix of plants (“barrier” height plants were added).

**Figure 14:**
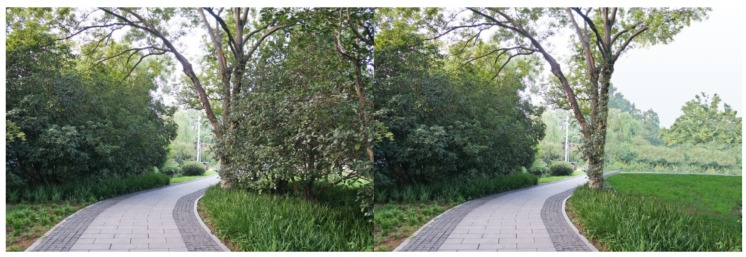
Visual accessibility (“screening” height shrubs at the right were removed, to reveal open view beyond).

Of the two photo pairs that did not show a statistically significant preference for the hypothesized image, one was an example of “Diverse mix of plants” in which a single type of shrub was added to the original photo (Figure 13), and the measure of preference was 53.36% (*p* = 0.259). The other was an example of “Visual accessibility” in which the screening vegetation was replaced by grass (Figure 14), and the preference for the hypothesized photo was 55.48% (*p* = 0.065). Appendix A, Table A1 shows the level of preference found for each of the 28 pairs of images tested in the study.

### 3.3. Gender Differences

**Figure 15:**
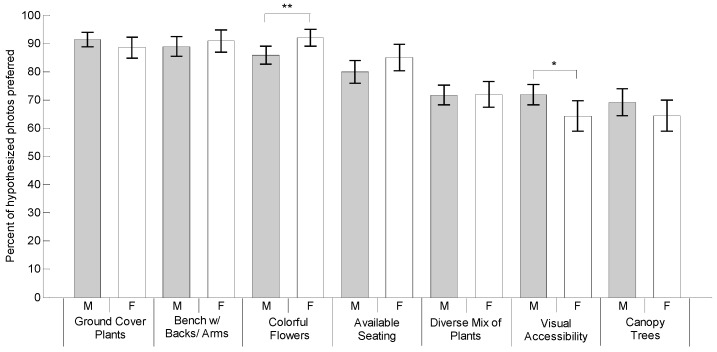
Mean with 95% confidence interval error bars for males and females.

On average, males chose the hypothesized preferred images 79.87% of the time, which was very similar to females (79.61%). As shown in Table 4 and Figure 15, males were more likely than females to choose the scenes depicting the construct “Visual accessibility” (*p* < 0.05); while females were more likely than males to choose the scenes with “Colorful flowers” (*p* < 0.01). 

### 3.4. Age Differences

**Figure 16:**
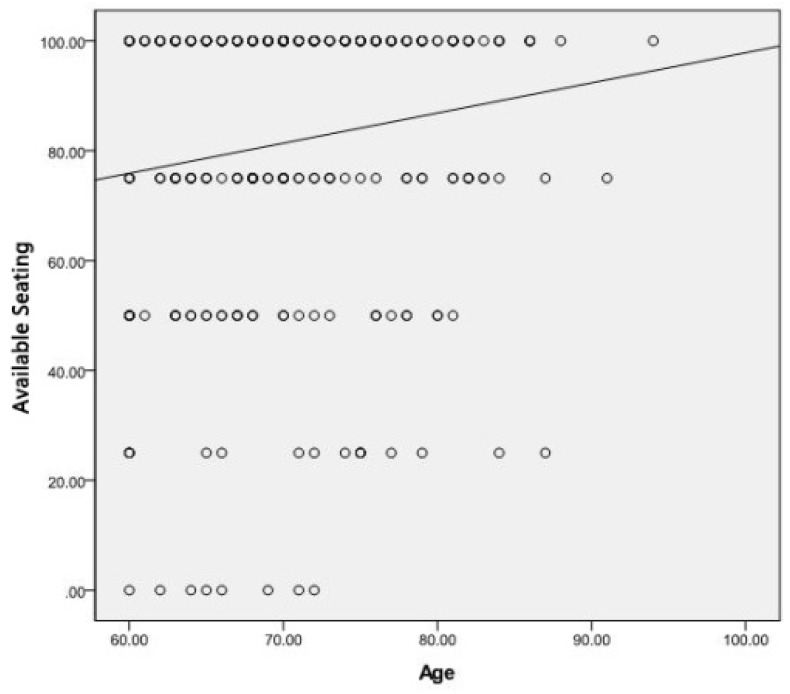
Scatter plot with fit line at total for ‘available seating’ by age.

A Spearman’s correlation was run to assess the differences in preference by age, treated as a continuous variable, and only a small positive correlation was found between age and the feature “Available seating” (*r* = 0.156, *p* = 0.009), indicating that preference for having benches available increased slightly with age (Figure 16). 

## 4. Discussion

### 4.1. Preferred Features

All seven hypothesized features were found to be significantly preferred by participants, suggesting that features such as seating, plant materials, and views along the walkways were perceived as important for satisfying older adults’ needs and supporting their park walking activities. The results generally support the relevant existing design guidelines for urban parks as well as the design recommendations developed especially for older adults [45,46,48,63,64,65,66,67].

The most preferred environmental characteristic was “ground cover plants,” which suggests older adults strongly preferred having continuous plant materials along the walkways, and disliked barren land with sparse vegetative cover. Participants also strongly preferred having seating available, and benches with backs and arms were even more highly preferred. The higher preference for benches found in older participants indicates an increasing need for benches with advancing age, allowing people to occasionally sit and rest while walking. These findings suggest that seating plays an important role in supporting older persons’ walking activity in urban parks. However, as we observed while taking photos, seating in most of the urban parks used in the study seemed inadequate to meet the needs of older adults; the problems included lack of seating, seating not placed in appropriate locations, and uncomfortable seating without backs and arms. While we tested only benches with wood seats in this study, stone and concrete benches were common in the parks; these have been reported in previous studies as being uncomfortable for older persons [68]. Since “colorful flowers” were so strongly preferred, these findings suggest that even small areas of herbaceous flowers can be valuable for older adults, allowing flowers to be planted in an economical and feasible way. The main gender difference in preferences was that male participants more strongly preferred "visual accessibility," while females more strongly preferred "colorful flowers." These differences might be due to differences in male and female visual perception, as males have been found to be more sensitive to detecting details from afar, and females better at distinguishing among colors [69,70].

The results of this study indicate that the way plants are used in the green space is also important, in addition to the actual viewable area of plant materials. For example, in testing the feature “diverse mix of plants” (Figure 7 and Figure 13), the compared photos showed almost the same amount of visible greenery; the main difference was the height of the plants. As another example, in the feature “visual accessibility,” the amount of visible greenery in the hypothetically-preferred photo was even less than in the non-hypothesized photo (Figure 8 and Figure 14). These examples suggest that preferences were likely due to having the plants arranged in a suitable way to support spatial hierarchy and human activities, rather than just having higher amounts of greenery. This finding may help designers understand how to appropriately locate plants in urban parks, to meet the needs of older users. 

Preference differences have been found among the pairs of image examples for each feature, such as “colorful flowers,” “diverse mix of plants,” and “visual accessibility” (Appendix A, Table A1). Subjects preferred the red and orange “colorful flowers” more than the example showing yellow flowers. It is also possible that flowers closer to eye level were preferred as they were more easily viewed than flowers in tall trees. Among the four paired photos representing the feature “diverse mix of plants,” only one hypothesized photo was not significantly preferred; this photo (Figure 13) showed a single type of small plant providing height diversity, while the other three hypothetically-preferred photos showed a variety or grouping of plants. It is possible that subjects preferred a walkway with diverse plants, but if the added plants were monotonous, they would not have a strong preference for it. In the images for “visual accessibility,” two photos had a view of a grassy area (e.g., Figure 14), and the other two photos had a view of water (e.g., Figure 8). Participants preferred the views of water more than those of grass; the measure of preference averaged 80.22% for water views, and 57.78% for grassy area views. These findings partially support previous research regarding the impact of blue space, which revealed that water features are generally preferred and have a positive effect on well-being [71,72].

### 4.2. Research Method and Instruments

Overall, the photographic comparison method used in this study was found to be a feasible and effective way to collect information from older adults in public settings. Most participants seemed happy to engage in the survey, completed it without assistance, and no one reported being fatigued or disinterested. 

### 4.3. Limitations and Future Research

While photographic comparison made it possible to study the landscape features tested in this study, not all features and site conditions can be clearly depicted in visual images, especially when taken from a single vantage point. For example, important features like lighting, walkway slope, and path layout also affect the use of urban parks but are less feasible to compare visually. Future studies could conduct behavior-mapping or in-depth interviews to better understand older people’s needs and environmental perceptions. One strength of this approach to photographic comparison is that each image pair isolates a specific feature. However, although efforts were made to provide diversity in the choice of images used in different photo pairs, by using dissimilar images to represent each feature, it is possible that different results would emerge if other images were presented; future studies could test these results by using additional images to represent urban park landscape features. Even the same types of features could be used in different colors or arrangements, which might result in different preferences being found. Due to time constraints, we surveyed only one district of the high-density urban area of Nanjing, but because the residents in this part of the city were similar to other districts in terms of race, ethnicity, and socio-economic status [56], the results are expected to be applicable to other densely populated districts in this city. Future studies could conduct additional analyses based on categories of participant age, ethnicity, etc. Since all the photos in this study were taken in summer, when the leaves were green, they do not depict seasonal variation. Future studies could explore preferences for landscape features across different seasons, using photographs taken in autumn, winter, or early spring.

## 5. Conclusions

By obtaining direct input from representative target users, this study provides empirical support for the value of several landscape features and principles already identified in the design literature. These concepts are expected to be useful in design practice and can be applied flexibly in a broad range of real-world settings. Since new land for constructing urban parks in high-density urban areas is limited and expensive, implementing these design principles in existing parks could help improve the quality of Chinese urban parks in a cost-effective manner. Providing enhanced walkway features can increase the usage of urban parks by older people, promoting walkability, physical activity, healthy aging, and potentially improving public health. As the world population continues to age, the findings of this study may also be adapted to the design and modification of high-density urban parks in other parts of the globe.

## Figures and Tables

**Table 1:** Mean age, number, and gender of participants in the six urban parks.

Urban Parks	Mean Age	Number and % of Participants	Males	Females
Xuanwu Lake Park	71.22 (6.68)	95 (33.57%)	65 (68.42%)	30 (31.58%)
Lovers Garden	71.57 (6.56)	51 (18.02%)	30 (58.82%)	21 (41.18%)
Pipa Continent	70.10 (7.53)	41 (14.49%)	25 (60.98%)	16 (39.02%)
Qian Lake	70.70 (7.68)	37 (13.07%)	19 (51.35%)	18 (48.65%)
Xiamafang Relics Park	70.47 (6.83)	32 (11.31%)	18 (56.25%)	14 (43.75%)
Sports Park	70.85 (6.52)	27 (9.54%)	19 (56.25%)	8 (43.75%)
Total	70.93 (6.88)	283 (100%)	176 (62.19%)	107 (37.81%)

Standard deviations of the mean age, percentages of the participants. and their gender in each urban park are shown in parentheses.

**Table 2:** Measure of preference for hypothesized features.

		95% Confidence Interval for Mean			
Landscape Features	Mean	Lower	Upper	Std. Error of Mean	*t*
Ground Cover Plants	90.37	88.24	92.50	1.08	37.37 ***
Bench w/ Backs/ Arms	89.66	87.05	92.28	1.33	29.91 ***
Colorful Flowers	88.25	85.92	90.58	1.18	32.35 ***
Available Seating	81.89	78.82	84.96	1.56	20.45 ***
Diverse Mix of Plants	71.82	69.07	74.57	1.40	15.61 ***
Visual Accessibility	68.99	65.91	72.08	1.57	12.12 ***
Canopy Trees	67.40	63.77	71.03	1.84	9.44 ***

One-sample *t*-tests at test value 50; *** *p* < 0.001.

**Table 3:** Mean differences and *p*-values for comparing preferences between features.

Landscape Features	Ground Cover Plants	Bench with Backs and Arms	Color Flowers	Available Seating	Diverse Mix of Plants	Visual Accessibility	Canopy Trees
Ground cover Plants	-	0.71 (0.642)	2.12 (0.159)	**8.48 (<0.001)**	**18.55 (<0.001)**	**21.38 (<0.001)**	**22.97 (<0.001)**
Bench with backs/arms	-	-	1.41 (0.360)	**7.77 (<0.001)**	**17.84 (<0.001)**	**20.67 (<0.001)**	**22.26 (<0.001)**
Colorful flowers	-	-	-	**6.36 (0.001)**	**16.43 (<0.001)**	**19.26 (<0.001)**	**20.85 (<0.001)**
Available seating	-	-	-	**-**	**10.07 (<0.001)**	**12.90 (<0.001)**	**14.49 (<0.001)**
Diverse mix of plants	-	-	-	-	-	2.83 (0.220)	**4.42 (0.024)**
Visual accessibility	-	-	-	-	-	-	1.59 (0.557)
Canopy Trees	-	-	-	-	-	-	-

Bold: mean difference reached the level of significance at *p* < 0.05.

**Table 4:** Gender differences in preference for hypothesized features.

Landscape Features	Mean Difference	Std. Error Difference	*t*	*p*
Ground Cover Plants	2.93	2.31	1.2682	0.2062
Bench with Backs/Arms	−1.97	2.74	−0.7187	0.4729
Colorful Flowers	−6.12	2.25	−2.7241	0.0069 **
Available Seating	−5.08	3.21	−1.5824	0.1147
Diverse Mix of Plants	−0.23	2.89	−0.0795	0.9367
Visual Accessibility	7.62	3.31	2.3002	0.0225 *
Canopy Trees	4.69	3.80	1.2350	0.2179

* *p*-value < 0.05; ** *p*-value < 0.01.

## Appendix A

**Table A1:** All of the 28 paired images (four pairs for each feature) and preference for each photo example.

			Mean	Std. Error Mean	*t*	*p*
	***Ground Cover Plants***					
Pair 1			86.93	2.01	18.394	<0.001
Pair 2			93.99	1.41	31.091	<0.001
Pair 3			92.58	1.56	27.280	<0.001
Pair 4			87.99	1.94	19.620	<0.001
	***Bench with Backs/ Arms***					
Pair 1			89.75	1.81	22.012	<0.001
Pair 2			90.11	1.78	22.556	<0.001
Pair 3			88.69	1.89	20.518	<0.001
Pair 4			90.11	1.78	22.556	<0.001
	***Colorful Flowers***					
Pair 1			91.17	1.69	24.360	<0.001
Pair 2			89.75	1.81	22.012	<0.001
Pair 3			83.04	2.23	14.784	<0.001
Pair 4			89.05	1.86	20.995	<0.001
	***Available Seating***					
Pair 1			87.99	1.94	19.620	<0.001
Pair 2			74.20	2.61	9.291	<0.001
Pair 3			78.09	2.46	11.405	<0.001
Pair 4			87.28	1.98	18.788	<0.001
	***Diverse Mix of Plants***					
Pair 1			53.36	2.97	1.130	0.259
Pair 2			83.04	2.23	14.784	<0.001
Pair 3			65.02	2.84	5.288	<0.001
Pair 4			85.87	2.07	17.289	<0.001
	***Visual Accessibility***					
Pair 1			68.20	2.77	6.562	<0.001
Pair 2			60.07	2.92	3.453	0.001
Pair 3			92.23	1.59	26.483	<0.001
Pair 4			55.48	2.96	1.851	0.065
	***Canopy Trees***					
Pair 1			65.02	2.84	5.288	<0.001
Pair 2			69.26	2.75	7.009	<0.001
Pair 3			66.78	2.80	5.984	<0.001
Pair 4			68.55	2.76	6.709	<0.001
